# The Role of Neuroergonomics in the Design of Personalized Prosthesis: Deepening the Centrality of Human Being

**DOI:** 10.3389/fnbot.2022.867115

**Published:** 2022-05-06

**Authors:** Laura Corti

**Affiliations:** Research Unit Philosophy of Science and Human Development, Department of Engineering, University Campus Bio-Medico of Rome, Rome, Italy

**Keywords:** neuroprosthesis, embodiment, neuroergonomics, design, neurophenomenology

## 1. From HRI to Human-Robot Merge

In recent decades, we have witnessed the rapid development of new technologies in several fields of our life. In particular, robotics grows so fast that it is often described as the technology of the future, as in Gates (2007); this trend is also confirmed by the Executive Summary World Robotics (IFR, 2020). From a theoretical point of view, all these devices could be framed according to a paradigm, which describes technology as a “medium” that relates the human being and the world (Ihde, 1990; McLuhan, 1994; Floridi, 2014); in this way, the technological devices could be depicted by two main characteristics: a) “being-between” (Floridi, 2014), and b) “being-for” (Heidegger, 2010).

The first dimension, the “in-betweenness,” describes the functional and practical role of technological mediation that takes place in the human-world relation; the preposition “between” identifies the physical mediation of the devices which play, in different ways (Ihde, 1990), the role of an intermediary of the experience. Instead, the second dimension, the “being-for,” emphasizes an opposite dynamic related to the relationship between human beings and technological devices. In this case, the preposition “for” highlights the necessity to design technology for someone and, for this reason, to consider the technology according to a defined setting and a specific condition. In particular, the “being-for” implies the necessity to rethink technological devices according to a human-friendly paradigm. In this emerging framework, the importance of a relational approach to technology becomes relevant in the design of reliable, efficient, and safe systems. Recently, this focus on the user and their needs has been deepened into the human-centered approach (Boy, 2017; Auernhammer, 2020); its importance can be found in all devices requiring the development of synergies and relationships between human and machine, from industrial robots to bio-medical devices (Riener et al., 2005; Schaal, 2007; Zhou et al., 2017).

This article will address the case of active upper-limb prostheses to discuss the importance and the limits of the neuroergonomics approach and human-centered design. In the relationship exemplified by prosthesis, the technological device physically alters the human being (Verbeek, 2008). From the theoretical point of view, this intimate relation opens up a new interaction model, which is based on a “merge” between the subject and technology. In line with Carrozza (2019), it is possible to argue that this form of mediation goes toward a neurophysiological symbiosis between humans and machines. The primary consequence of this approach is a focus on neurophysiological aspects considered essential and irreducible. Nevertheless, this article argues that this emphasis is unable to gather the dimensions necessary to develop functional and accepted prostheses. In particular, this opinion paper argues that a neuro-based approach is a necessary but not sufficient requirement for a human-friendly device.

## 2. Neuro-approaches: Benefits and Risks

In response to the need to design prostheses “for” human beings, which have better functionality and controllability (Carrozza et al., 2006; Zollo et al., 2007; Atzori and Müller, 2015), research and prototypes of bio-inspired artificial limbs have been developed in recent years. Compared to cosmetic prostheses, these active devices, which are able to manipulate objects, have a greater degree of usability, significantly improving users' quality of life and ADL (Activities of Daily Living) (Cordella et al., 2016). Mainly the manipulative capacity is modeled through myoelectric control (Scott and Parker, 1988), or neural interface (Schultz and Kuiken, 2011). The myoelectric control is based on identifying user intention through MES (myoelectric signals) (Geethanjali, 2016); instead, in most cases, the neural interfaces use TIME (Transversal Intrafascicular Multichannel Electrode) (Badia et al., 2015). In this regard, a recent study described the use of the PNS (peripheral nervous system) as an “elegant” strategy to improve HRI because “peripheral neural information can complement psychometric and physiological methods to assess user experience, which indicates that integrating myographic assessment in multimodal PNS-MIs would bring the neuroergonomics of human-robot interaction to a new level of quality.” (Del Vecchio et al., 2021). Both these methodologies exploit the PNS (Ciancio et al., 2016) that is emerging as the “ideal channel” of human-robot interaction (Del Vecchio et al., 2021). The methods mentioned above realize this “merge” according to two opposite dynamics.

The use of myoelectric technologies has had a significant development in recent years because of some advantages that are recognized by it. The decoding of the MES takes place in a non-invasive way, as it is detected through the surface of the skin, and is a reasonably accurate way as little muscle activity is needed to control the prosthesis (Parker et al., 2006). Currently, this method of prosthesis control is the most widely used for commercial purposes. The TIME, on the contrary, is a more invasive technique that allows not only to decode the intention of the user but also to return manipulation feedback. For this reason, this methodology needs electrodes implanted in the afferent and efferent pathways (Raspopovic et al., 2014) for bidirectional control of the prosthesis. Although they are still investigational and very invasive techniques, early studies, as in Zollo et al. (2019), highlight that, compared to myoelectric technology, bidirectional prostheses have more refined control in grasping and manipulating objects. This is explicitly due to the sensory capacity of the prosthesis.

Since the literature review (Cordella et al., 2016) has revealed a better control of the prosthesis and a more remarkable ability to manipulate these devices, it is evident the strong appeal that the neuro-based approach has in the design of efficient prostheses. From this perspective, it is possible to affirm the central role of neuroergonomics for further investigation focused on the user's needs. In particular, this discipline, which studies the brain and its functions in performing tasks (Parasuraman, 2003; Parasuraman and Wilson, 2008), has a strong impact in the field of biomedical engineering and prosthesis design because neuroergonomics defines an innovation working on the deep investigation of perceptual and cognitive functions (Parasuraman, 2003). This approach, based on recognizing the brain's role in perception, highlights the quantitative measure of the stimulus and its reproduction in an artificial system. This approach is helpful in developing systems that are able to realize a synergic “merge” between human beings and technological devices; the significant benefit of neuroergonomics can be summarized in two main aspects: a) the discovery of the neural basis of perception, and b) a more careful analysis of the neural resource of the action, such as grasping. For this reason, in line with Parasuraman (2003), I recognize an “added value” for this research field that goes beyond the traditional limits of both neuroscience and the ergonomic approach. According to this statement, it is possible to conclude that there are several advantages to using a neuro-based approach in terms of prostheses functionality and performance; indeed, a neuroergonomics study could have finer decoding of the user's intention based on the brain activity and better comprehension of the manipulation and grasping tasks.

In conclusion, the “added value” of neuroergonomics concerns, in particular, the chance to design and development of more functional and personalized prostheses. In this perspective, the lack of functionality is recognized as a factor hindering the use of the prosthesis (Biddiss and Chau, 2007); for this reason, it (Cook and Polgar, 2015) is conceived as a necessary element. Scientific evidence, as Petrini et al. (2019), supports the hypothesis that an approach based on the body's neurophysiology represents a valuable solution to the problems currently plaguing commercial prostheses. Nevertheless, we may question whether a neuro-based approach is capable of guiding design through a comprehensive focus on the human being. In this perspective, functionality understood as the method that estimates the performance of the device (Chappell, 2016), turns out to be a necessary but not sufficient condition for a human-friendly device as this perspective lacks in considering the consequences and reasons that lead subjects to refuse or reject prostheses. In literature, it is possible to find alternative solutions that try to solve the problem; e.g., Biddiss proposes a human-centered approach, called Need-Directed Design, which provides a study of prostheses according to the priorities of the user able to take into account, specifically, comfort, cost, anthropomorphism, sensation, and functionality (Biddiss, 2009). Starting from this approach, it is possible to identify another useful parameter for the design of prostheses, the first-person experience^1^. It concerns a direct stakeholder's involvement in the design phase of the prosthesis. The analysis of the first-person prosthesis experience is not intended to replace the neuro-based approach but rather to support it by making explicit the central role of the user. This integration responds to the problem of the explanatory gap (Levine, 1983) by trying to address neuro-based and phenomenological approaches as two different perspectives on the subject. From a methodological point of view, this new approach, which can be defined as quanto-qualitative (Corti, 2021), wants to combine diverse perspectives. This new investigation relates both objective data, obtained by measuring the stimulus, and the subjective feeling, described during the prosthesis's use. This critique aims not to revoke in doubt the central role of the brain in the design phase but rather to highlight the incomplete adequacy of a neuro-based paradigm for personalized devices. Specifically, as argued before, a direct user's involvement in the design phase can significantly improve prosthesis acceptance.

## 3. Discussion: A Human-centric Approach, Including the First-person Dimension in the Design of Prostheses

In the design of personalized prostheses, the phenomenological dimension that involves the first-person approach is becoming increasingly important; e.g., Biddiss explicitly states, “If a person feels that a prosthesis enhances their function and/or appearance, they will use the device. Conversely, if the prosthesis is perceived to hinder function or comfort, or spoil the appearance, they will not use the device” (Biddiss, 2009). Therefore, recognizing the importance of feeling for prosthetics implies the need to rethink an appropriate methodology, which includes a phenomenological dimension, for assessing prosthetic acceptability and embodiment. It is clear that even if a neuro-based approach allows the creation of interfaces between computer and brain, a first-person analysis also has significant benefits for prosthetic design. Specifically, this new methodology helps investigate upper limb prostheses with haptic feedback as the sensory feedback implies the first-person dimension. For this reason, in the evaluation of sensitive prostheses, direct involvement of patients' subjective reports is mandatory, as in Zollo et al. (2019). Nevertheless, from a methodological point of view, there are two potential risks:

1. consider subjective reports as secondary in that they are useful only to support neuroscientific findings;
2. not investigating the experience according to a rigorous methodology and criteria.

In literature, it is possible to find some methods that solve the above problems and integrate the two dimensions, e.g., neurophenomenology (Varela, 1996; Lutz and Thompson, 2003). Specifically, this approach aims at emphasizing how a first-person approach can provide additional and essential information (Thompson and Cosmelli, 2005) for the neuroscientific investigation^2^.

The neurophenomenological approach has been empirically tested in some studies, such as Lutz et al. (2002), Lutz (2002), Lutz and Thompson (2003), and Lutz et al. (2008). In particular, Lutz et al. (2002) conducted a study on visual tasks in which, in front of continuous monitoring through Electroencephalography (EEG), the subject is asked to describe the phenomenological content of the action performed. The study showed that it is possible to establish a relationship between the subject's verbal descriptions and the measurement of neural activity. The recognition of mutual constraints between first-person experience and EEG data suggests that the same study can be applied to upper limb prostheses. This quanto-qualitative approach involves the subject in the design process in an active and participatory^3^ way (Corti et al., 2020). In this perspective, it is plausible to hypothesize experimental settings to find mutual constraints between the subjective (qualitative) reports on manipulation tasks and the quantitative measure of brain activity. In particular, this strategy aims at highlighting some phenomenological elements relevant to personalized prostheses, such as naturalness of sensation, perceived ability, and embodiment.

In conclusion, I argue that the mixed paradigm proposed above can help in the development of functional devices and also in detecting prosthetics embodiment. Thus, the quanto-qualitative approach helps to connect paradigms, e.g., the phenomenological and the neuroscientific ones, shedding light on issues, such as embodiment. Adopting a methodology capable of integrating multimodal data supports the investigation of embodiment since it has 2-fold nature and cannot be completely quantified (Corti, 2021). On one side, it has a neurophysiological basis; on the other side, it is a phenomenological dimension (Murray, 2008; De Preester and Tsakiris, 2009; De Preester, 2011). Specifically, three conditions seem to emerge that simultaneously involve the neurophysiological aspect and the phenomenological dimension: (a) the physical presence of the prosthesis in continuity with the body, (b) the disposition to use the prosthesis for action, and (c) the recognition of that device as part of one's body.

## Author Contributions

LC conceived of the study and drafted the manuscript.

## Conflict of Interest

The author declares that the research was conducted in the absence of any commercial or financial relationships that could be construed as a potential conflict of interest.

## Publisher's Note

All claims expressed in this article are solely those of the authors and do not necessarily represent those of their affiliated organizations, or those of the publisher, the editors and the reviewers. Any product that may be evaluated in this article, or claim that may be made by its manufacturer, is not guaranteed or endorsed by the publisher.

## Footnotes

^1^In this perspective, I argue that the inability to take into account the first-person experience of the prostheses use may afflict the phase of personalization of the prosthesis.

^2^In line with Gallagher and Zahavi (2020), I argue that this new research methodology can offer a direct contribution to the empirical research and, specifically, to the prostheses design developing a more accurate evaluation of Human-Robot Interaction.

^3^From a methodological point of view, the subject's involvement in the design phase contributes to the opening of the human-centered approach in a human-centric paradigm. On the one hand, the human-centered approach emphasizes the user's perspective, which, as we have seen in the case of prostheses, may imply the measurement of the stimulus; on the contrary, the emerging human-centric involves directly the stakeholders. In the case of bionic prosthesis design, the shift between human-centered and human-centric paradigm can be represented by a third-person paradigm, which evaluates as relevant only the objective measure of brain stimulus, and a phenomenological perspective that, directly involving the subject through a questionnaire or personal reports, also captures the first-person dimension of experience.
